# Global constitutionalism, responsibility to protect, and extra-territorial obligations to realize the right to health: time to overcome the double standard (once again)

**DOI:** 10.1186/s12939-014-0068-4

**Published:** 2014-10-10

**Authors:** Gorik Ooms, Rachel Hammonds

**Affiliations:** Department of Public Health, Institute of Tropical Medicine, Antwerp, Belgium; School of Public Health, University of Western Cape, Cape Town, South Africa; Law and Development, Faculty of Law, University of Antwerp, Antwerp, Belgium

**Keywords:** Human rights, Right to health, Global constitutionalism, Responsibility to protect, Extra-territorial obligations

## Abstract

If human rights are “inalienable rights of all members of the human family”, as is enshrined in the Universal Declaration of Human Rights, then no government should be allowed to deny people of them. When some governments fail to realize them for the people under their jurisdiction, the international community has a responsibility to step in.

This extra-territorial effect of human rights was not included in the original conception of human rights. It is of recent date, and, in practice, limited to interventions to end severe violations of civil and political human rights. For economic, social and cultural human rights, extra-territorial obligations are still contested.

In this paper, we elaborate three contentions: first, that the realization of social human rights requires the acceptance of and compliance with extra-territorial obligations; second, that compliance with extra-territorial obligations would help transform the international assistance paradigm from charity into legal obligation; and third, that for global constitutionalism to succeed in improving the fairness of the international legal order requires acceptance of the indivisibility of human rights.

## Introduction

If human rights are “inalienable rights of all members of the human family”, as enshrined in the Universal Declaration of Human Rights (UDHR) [1], then no government should be allowed to deny people of them – whether the denial is due to the inability or the unwillingness of some governments to realize all human rights for all people under their jurisdiction. If, for practical and historical reasons, it made sense to assign the duties corresponding with human rights primarily to the government that rules the territory where the people concerned live, this assignment or designation cannot be exclusive. If the designation of corresponding duties were exclusive, then the realization of human rights would depend entirely on the discretionary willingness or the fortuitous ability of state governments – and that would make human rights very alienable indeed. In the words of Shue: “where the state with the primary duty to protect rights fails – for lack of will or capacity – to fulfill its duty, some other agent at least sometimes must step in and provide the missing protection”, [2] pages 176–177.

The original conception of human rights did not include such ‘extra-territorial’ responsibility. The (French) Declaration of the Rights of Man and Citizen of 1789 – considered by Cohen as the “progenitor and referent of modern human rights discourses” [3], page 166 – was, as its title suggests, a declaration about the rights of the citizens of France. While the text may suggest that all human beings ought to enjoy the rights mentioned in the declaration, it does not suggest that the people of France rely on assistance from abroad for their enjoyment, or that France should promote these rights elsewhere: these are citizens’ rights – rights one has by virtue of being a citizen of a society that embraced them – rather than human rights.

According to Cohen, a “second wave of human rights discourse and treaty-making” was marked by “the invocation of internationally and regionally articulated human rights agreements by local activists” [3], pages 168–169: a passive form of extraterritorial responsibility for human rights. By passive, we mean that the international community set human rights standards for all countries, but relied on activism at the national level claiming their realization, and ultimately on governments to fulfil them at the national level. Cohen situates the UDHR at the end of the ‘first wave’ of human rights discourse – because it seems to confirm national sovereignty unconditionally – whereas we would consider the UDHR as the starting point of the ‘second wave’ – a retroactive rejection of the abuses of sovereignty during World War Two, setting standards for all countries – we can agree that human rights discourse took an important turn. Importantly, international legal scholars broadly agree that the UDHR now forms part of customary international law, and although not a legally binding treaty it establishes universal standards to be upheld and realized for all [4]. Under the ‘second wave’, human rights are no longer perceived as rights that *some* people can claim from their governments – depending on whether their governments decide to embrace them, or not; they have become rights that all people can legitimately claim from their governments – whether governments embrace them or not. But under the ‘second wave’ the responsibility to realize human rights still rests at the national level.

Cohen argues that a ‘third wave’ of human rights discourse started when human rights were “invoked as justification for the imposition of debilitating sanctions, military invasions, and authoritarian occupation administrations by multilateral organizations (NATO, UNSC) and/or states acting unilaterally under the rubric of “humanitarian” and even “democratic” intervention” [3], page 172. This is what we would call an active form of extraterritorial responsibility for human rights: when governments fail to realize them, the international community has a responsibility to step in.

The so-called ‘responsibility to protect’ (R2P) doctrine, embraced by limited elements of the international community in recent years [5], can be seen as the spearhead of the third wave of human rights. The R2P doctrine is summarized by Bellamy as resting “on three equally weighted and nonsequential pillars:

1. the primary responsibility of states to protect their own populations from the four crimes of genocide, war crimes, ethnic cleansing, and crimes against humanity, as well as from their incitement;
2. the international community’s responsibility to assist a state to fulfill its R2P; and
3. the international community’s responsibility to take timely and decisive action, in accordance with the UN Charter, in cases where the state has manifestly failed to protect its population from one or more of the four crimes” [5].

While it is difficult to argue against the idea that the international community ought to take action when governments fail “to protect their own populations from the four crimes of genocide, war crimes, ethnic cleansing, and crimes against humanity” – which most often means governments involved in genocide, war crimes, ethnic cleansing, and crimes against humanity – we find the present narrow scope of the ‘third wave’ of human rights discourse deeply problematic. Others have criticized the arbitrary application of the R2P doctrine: at times it is applied, at times it is not, and whether it is or not may depend more on political factors than on objective human rights assessments [6]. While we share this critique, the one we want to develop here is of a somewhat different nature: that the ‘third wave’ of human rights discourse is selective, focusing on severe violations of civil and political human rights (we will call them political human rights in this paper), while turning a blind eye to gross violations of economic, social and cultural human rights (we will call them social human rights in this paper).

In the area of social human rights the concept of ‘Extra-Territorial Obligations (ETOs) of States in the Area of Economic, Social and Cultural Rights’ shares some features with the R2P concept. The parameters of the ETOs concept were codified in an authoritative list by experts in international law and human rights at a meeting convened by Maastricht University and the International Commission of Jurists [7]. Unlike the R2P concept, which has been “ adopted unanimously by heads of state and government at the 2005 UN World Summit and reaffirmed twice since by the UN Security Council” [5], the ETOs concept is still in its infancy. As we will elaborate further below, the international community seems rather reluctant to embrace the idea that extra-territorial responsibility for human rights could include social human rights as well.

To be clear, we believe in the potential merits of so-called ‘global constitutionalism’ – in the words of Peters, “the academic and political agenda that identifies and advocates for the application of constitutionalist principles in the international legal sphere in order to improve the effectiveness and the fairness of the international legal order” [8] – and we believe that international human rights law should be the cornerstone of global constitutionalism. But we are critical proponents; we argue that if the human rights discourse on extraterritorial responsibility remains focused on political human rights, excluding social human rights, global constitutionalism will be “seen purely as a liberal project whose overriding goal, though not explicitly stated, is the imposition of Western-style liberal democracy, complete with its condiments” [9].

In this paper, we elaborate three contentions:

- First, that the realization of social human rights requires the acceptance of and compliance with ETOs;
- Second, that compliance with ETOs would start to shift the international assistance paradigm from charity into legal obligation, and that this provides a plausible explanation for the international community’s reluctance to embrace ETOs;
- Third, that for global constitutionalism to “improve the effectiveness and the fairness of the international legal order” [8] – it needs to accept the indivisibility of human rights – political *and* social human rights, R2P *and* ETOs.

### Why ETOs matter for social human rights

A comprehensive overview of all ETOs for all social human rights would not fit within a single paper. We will discuss one particular ETO for one particular social right: the obligation to provide international assistance for the realization of the right to health. This is not to suggest that the right to health is the most important social human right; it is one that allows us to illustrate the deadly consequences of rejecting ETOs. And this is not to suggest that providing international assistance is a sufficient condition for the realization of the right to health; it is but one of the essential conditions, at least in some countries.

The basic argument is straightforward: if a health system able to deliver essential healthcare services costs at least US$55 per person per year, at least some low income countries cannot afford that, even if they tried hard. But if high-income countries would allocate the equivalent of 0.1% of their gross domestic product (GDP) to international assistance for health, in addition to low and middle income countries making progress towards allocating at least 5% of GDP to public health expenditure, then essential healthcare services could be delivered in all countries [10].

Like many other international human rights scholars, we consider the text of article 2 (1) of the International Covenant on Economic, Social and Cultural Rights, as an acceptance of an ETO to provide international assistance. We agree with Tobin when he writes that this ETO has “a solid textual foundation under article 2(1)”, but also when he writes that “the scope and the nature of this obligation remain contested” [11]. Although the textual foundation is solid, the accountability mechanisms are weak; governments of high income countries cannot be brought before an international court for not living up to their ETOs. As long as these governments refuse to live up to these duties in practice, any confirmation that they have accepted them in theory will remain controversial.

Another foundation of the ETO to provide assistance is the promise high income countries made in 1970, to allocate the equivalent of 0.7% of their GDP to official development assistance (ODA) [12]. ODA is not exclusively intended to realize social human rights, but it is “administered with the promotion of the economic development and welfare of developing countries as its main objective” [12], and thus we can qualify the commitment to allocate the equivalent of 0.7% of GDP to ODA as an acceptance of the ETO to provide assistance. Furthermore, as Khalfan argues, “[The International Covenant on Economic, Social and Cultural Rights] does not give any guidance as to the proportions of resources available for the [International Covenant on Economic, Social and Cultural Rights] that a State must utilise for territorial and extraterritorial obligations, respectively” [13]. And he continues arguing that in addition to a ‘process criterion’ (a fair and honest assessment of a state’s ability to fulfil social human rights extraterritorially), three substantive criteria could be used, namely “whether a State has achieved internationally agreed benchmarks and unilateral commitments”, “whether steps taken are reasonable in comparison to peer States”, and “whether the State has progressively increased the extent of its assistance and cooperation as its available resources increase” [13]. We would argue that the 0.7% of GDP norm is such an ‘internationally agreed benchmark’ , and that allocating 15% of ODA (or 0.1% of GDP of high income countries) is ‘reasonable in comparison to peer States’.

One could argue that the international community is *de facto* living up to its ETO to provide assistance, without explicitly acknowledging the ETO. After all, many countries made progress in the realization of the right to health, long before acknowledging the right to health explicitly. Perhaps this is what the international community is doing: complying with its ETO to provide assistance, without acknowledging it explicitly. But this is problematic for a number of reasons.

First, ODA that is not based on an acknowledged ETO creates a donor and recipient relationship, rather than a duty-bearer and rights-holder relationship. As the former UN Special Rapporteur on the Right to Health expressed it: “if there is no legal obligation underpinning the human rights responsibility of international assistance and cooperation, inescapably all international assistance and cooperation is based fundamentally upon charity. While such a position might have been tenable 100 years ago, it is unacceptable in the twenty-first century” [14].

Second, ODA that is based essentially upon charity is unreliable. Foster observes that “donor disbursement performance remains volatile and unreliable”, and that “governments are therefore understandably reluctant to take the risk of relying on increased aid to finance the necessary scaling up of public expenditure” [15]. As a result, public health policies for low income countries are generally based on the assumption that funding for recurrent expenditure should not be based on ODA. This is the main reason why, for example, Marseille and colleagues argued that HIV prevention should be given priority over AIDS treatment [16], or why Costello and colleagues challenged the strategy to reduce maternal mortality that was based on increasing access to basic emergency obstetric care in health centers [17].

Third, complying with an ETO – even partially – without acknowledging the ETO can be perceived as rejecting the ETO. This explains why, in 1990 – at the beginning of the ‘third wave’ of human rights discourse – Mahbubani – then a Singaporean diplomat in Washington DC, now Dean of the Lee Kuan Yew School of Public Policy of the National University of Singapore – described how many citizens of poorer countries felt about Western preoccupation with select human rights [18]:

“They are like hungry and diseased passengers on a leaky, overcrowded boat that is about to drift into treacherous waters, in which many of them will perish. The captain of the boat is often harsh, sometimes fairly and sometimes not. On the river banks stand a group of affluent, well-fed, and well-intentioned onlookers. As soon as those onlookers witness a passenger being flogged or imprisoned or even deprived of his right to speak, they board the ship to intervene, protecting the passengers from the captain. But those passengers remain hungry and diseased. As soon as they try to swim to the banks into the arms of their benefactors, they are firmly returned to the boat, their primary sufferings unabated.”

### The return of an old division

Soon after agreeing the UDHR [1], the ramping up of the Cold War saw the corpus of human rights cleaved in two. The West championed political human rights; the former Eastern Bloc and the Non-Aligned Movement championed social human rights. Each part was further elaborated in a separate treaty: the International Covenant on Civil and Political Rights [19], and the International Covenant on Economic, Social and Cultural Rights [20].

This division goes back to an older division of rights into ‘negative’ and ‘positive’ rights. Negative rights, entail duties to refrain from doing something, i.e. for, freedom of expression the main corresponding duty for society is to not interfere when a person wants to express her or his opinion. The right to health is considered a positive right, because the corresponding duty for society is to make efforts to protect or promote a person’s health. This is the main reason why some scholars argued that only civil human rights – considered negative rights – could be ‘real’ human rights. Cranston, for example, argued [21], page 66:

“The traditional ‘political and civil rights’ can (as I have said) be readily secured by legislation; and generally they can be secured by fairly simple legislation. Since those rights are for the most part rights against government interference with a man’s activities, a large part of the legislation needed has to do no more than restrain the government’s own executive arm. This is no longer the case when we turn to ‘the right to work’, ‘the right to social security’ and so forth. For a government to provide social security it needs to do more than make laws; it has to have access to great capital wealth, and many governments in the world today are still poor.”

The weakness of the logic underpinning this rigid division is readily apparent; for example, our freedom to express our opinions in this paper may depend on public positive efforts like protection against aggression from people who disagree with our opinions, while our right to health may depend on public negative efforts like preventing the burning of toxic waste. Furthermore, as many eminent scholars have argued before us, freedom of expression can be quite meaningless for people who are starving [2], while people living in societies where freedom of expression is limited may be at higher risk of famines and starvation [22].

If the “separation wall” between political and social human rights has been deconstructed in human rights discourse at the national level [23], it still stands firmly when it comes to extraterritorial obligations: R2P is accepted by the UN General Assembly, ETOs are not, and most high income countries reject them [11].

Human rights have assumed a prominent role in international relations, but that is truer for political human rights [24] than for social human rights [25]. This is surprising if we compare article 2(1) of the International Covenant on Civil and Political Rights with its mirror article in the International Covenant on Economic, Social and Cultural Rights. With regards to political human rights, states made a commitment to “respect and to ensure them to all individuals within its territory and subject to its jurisdiction” [19], whereas, with regards to social human rights, states made a commitment to realize them progressively, “individually and through international assistance and co-operation, especially economic and technical, to the maximum of their available resources” [20]. One would expect international relations to focus on social human rights, for which the corresponding duties are explicitly shared – in the language of the treaty. The origins of the division between political and social human rights may explain the focus on political human rights in international relations. For political human rights, with their corresponding duties considered to be negative and therefore free of costs, international cooperation can be expected to be limited to telling other governments what they ought to do, while for social human rights, with their corresponding duties considered to be positive and therefore costly, international cooperation required financial assistance.

### Global constitutionalism: the ‘imposition of western-style liberal democracy’ or ‘minimal decency’?

If the promise of global constitutionalism – “to improve the effectiveness and the fairness of the international legal order” [8] – is to be fulfilled, global constitutionalism’s proponents will have to consider the enormous health inequalities between so-called ‘developed’ and ‘developing’ countries. For example, Amouzou and colleagues recently found approximately 6.6 million under-five deaths in 2007 in the 67 developing countries they analyzed, and that “this could be reduced to only 600,000 deaths if these countries had the same under-five mortality rate as developed countries” [26], but also that “ if the under-five mortality rate was lowered to the rate among the top 10% economic group in each of these countries, under-five deaths would be reduced to 3.7 million” [26]. In our opinion, both the inequality within developing countries and between developed and developing countries constitute human rights violations. An international legal order that does not qualify them as such is neither fair nor effective.

In a recent paper, Frenk and colleagues argue that “the moment is ripe to revisit the idea of global health”, and their revised idea of global health would be based on “a shared commitment to realisation of health as a human right based on a recognition of our common humanity” [27]. We could not agree more, except in as much as the title of their paper – “From sovereignty to solidarity: a renewed concept of global health for an era of complex interdependence” – may suggest a complete departure from national sovereignty. To be sure, that is not what they imply; what they do imply looks very similar to the notion of ‘conditional sovereignty’ as advanced by Shue: “The basic idea is that states should have to behave with minimal decency if they want respect. Sovereignty should be conditional upon performance, and performance should be judged by international norms: *conditional sovereignty*, judged by minimum international standards, including the provision of protection for basic rights” (emphasis in original) [2], pages 174–175.

Taking ETOs seriously would affect the sovereignty of all countries: for example, high income countries would no longer be free to decide themselves how much assistance they would provide to which countries; some kind of coordination and accountability mechanism, agreed following extensive negotiation, would provide guidance. But this minimal limitation on sovereignty would be in line with ‘minimal decency’, considering the lives at stake, and the relatively modest effort required. A global constitutionalism project that is similarly demanding for all countries – and that ensures at the very least universal health coverage anchored in the right to health [28] – would have a much greater chance of succeeding than one that is perceived as the imposition of Western-style liberal democracy.
